# Supplementary material for: KLF7 orchestrates hippocampal development through neurogenesis and Draxin-mediated neuronal migration
Source: Development. 2025 Aug 5;152(20):dev204718. doi: 10.1242/dev.204718 (PMC12377812; doi:10.1242/dev.204718)
Supplement: Supplementary information [file develop-152-204718-s1.pdf]

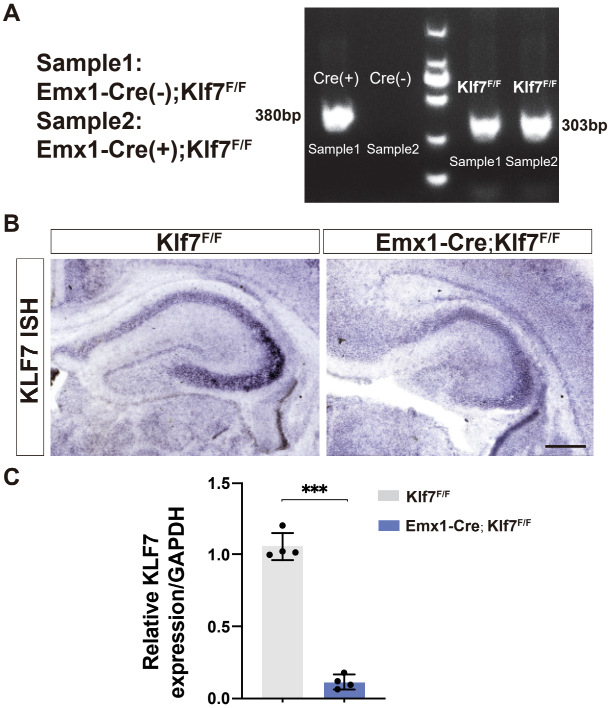

Fig. S1. Efficient KLF7 deletion in the hippocampus of Emx1-Cre;Klf7$^{F/F}$ mice.(A) PCR genotyping of Klf7$^{F/F}$ (control) and Emx1-Cre;Klf7$^{F/F}$ (cKO) mice. (B) In situ hybridization for Klf7 in P7 hippocampal sections. Scale bar, 100 μm. (C) RT-qPCR confirming reduced Klf7 expression in P1 hippocampus (n = 4 mice). Data are mean ± SEM; ***p < 0.001.

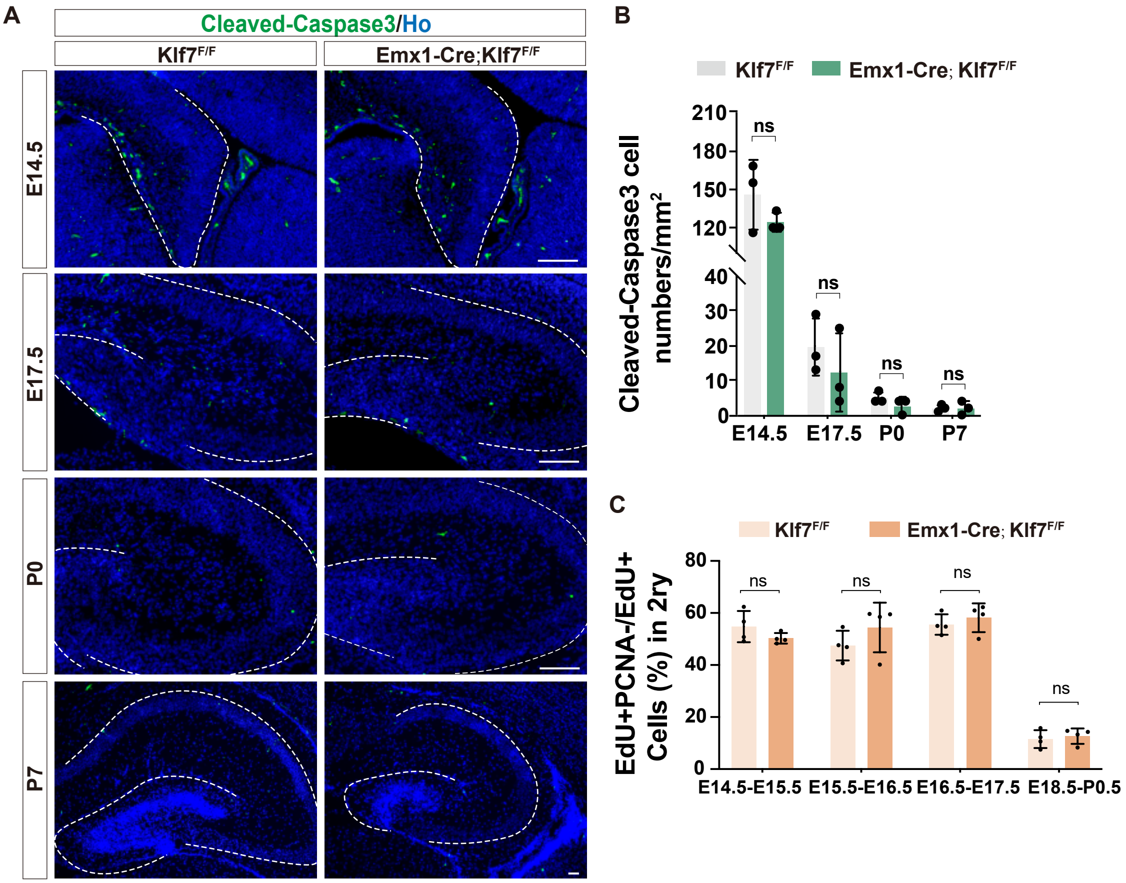

Fig. S2. KLF7 deficiency does not cause abnormal apoptosis in the hippocampus or affect progenitor cell cycle exit in the secondary germinative matrix.

(A) Immunofluorescence staining for cleaved caspase-3 at E14.5, E16.5, E17.5, P0, and P7 in brain sections from Klf7$^{F/F}$ and Emx1-Cre;Klf7$^{F/F}$ mice. Dashed lines outline the developing dentate gyrus (DG) region. Scale bar, 100 µm.

(B) Quantification of cleaved caspase-3^{+} cells in the hippocampus at E14.5, E17.5, P0, and P7 (n = 3 mice).

(C) Quantification of EdU^{+}PCNA^{-}/EdU^{+} cells in *Klf7$^{F/F}$* and *Emx1-Cre;Klf7$^{F/F}$* mice (n = 4 mice). ns, not significant; unpaired two-tailed t-test. Data are presented as mean ± s.e.m.

Development • Supplementary information

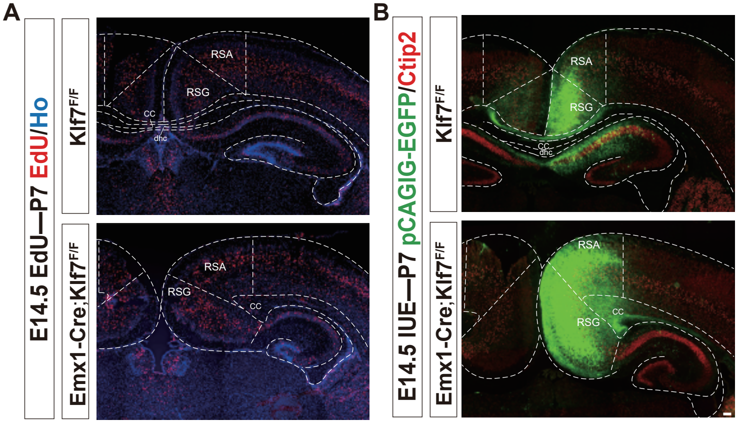

Fig. S3. Aberrant neuronal migration to the retrosplenial granular cortex (RSG) in KLF7 cKO mice.(A) EdU staining of P7 brain sections from Klf7$^{F/F}$ and Emx1-Cre;Klf7$^{F/F}$ mice injected with EdU at E14.5. Dashed lines delineate cortical and hippocampal regions. Scale bar, 200 μm. (B) Ctip2 immunostaining of P7 brain sections from Klf7$^{F/F}$ and Emx1-Cre;Klf7$^{F/F}$ mice electroporated with pCAG-IRES-EGFP.

Abbreviations: RSA, retrosplenial agranular cortex; RSG, retrosplenial granular cortex; CC, corpus callosum; dhc, dorsal hippocampal commissure.

Development • Supplementary information

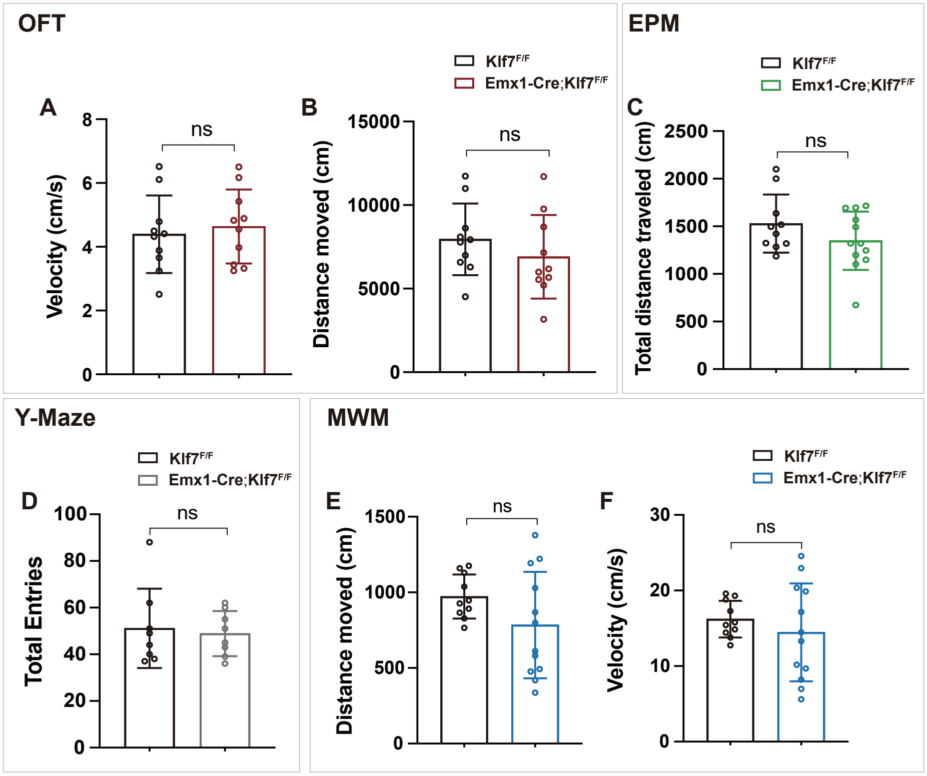

Fig. S4. Behavioral tests indicate normal motor function in Emx1-Cre;Klf7$^{F/F}$ mice.
(A-B) Open field test (OFT) results for Klf7$^{F/F}$ (n = 10) and Emx1-Cre;Klf7$^{F/F}$ mice (n = 10) at 6–8 months of age. Velocity (A), Total distance moved (B).
(C) Total distance traveled in the elevated plus maze test (EPM) for Klf7$^{F/F}$ (n = 10) and Emx1-Cre;Klf7$^{F/F}$ mice (n = 12) at 6–8 months of age.
(D) Total entries recorded during the Y-maze test for Klf7$^{F/F}$ (n = 8) and Emx1-Cre;Klf7$^{F/F}$ mice (n = 8) at 6–8 months of age.
(E-F) Morris water maze test (MWM) results for Klf7$^{F/F}$ (n = 10) and Emx1-Cre;Klf7$^{F/F}$ mice (n = 12) at 6–8 months of age. Distance moved (E), velocity (F).
Data are represented as mean ± SEM; ns, not significant.

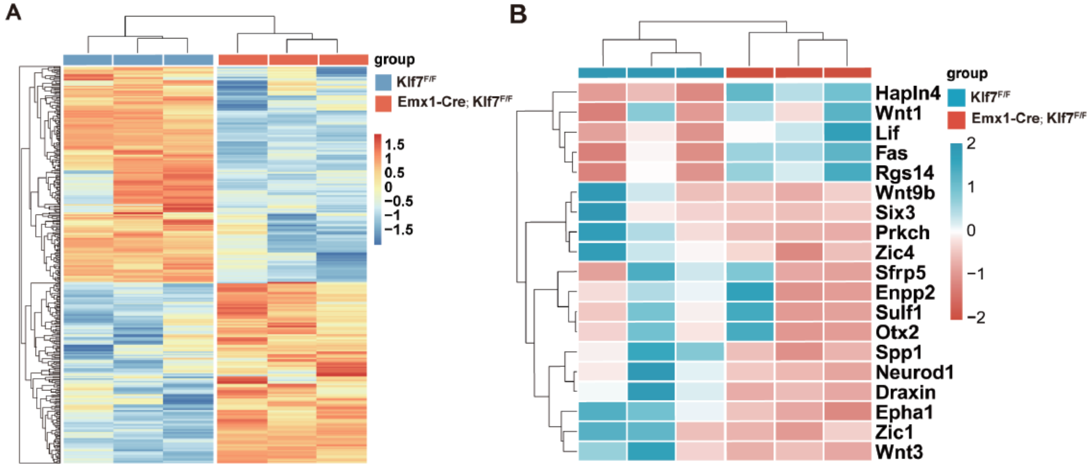

Fig. S5. Heatmap of differentially expressed genes (DEGs) by RNA-seq.

(A) Heatmap showing all significant DEGs identified in RNA-seq analysis (n = 3 mice).

(B) Heatmap highlighting DEGs involved in the regulation of neurogenesis (n = 3 mice).

Development • Supplementary information

Table S1. Primers (*In situ hybridization*, *PCR, RT-qPCR, Luciferase reporter assay, plasmids, Electroporation plasmids*)

Name	Base
In situ hybridization	
iKLF7-Forward	ACCTGCCTCAGCCTCCAG
iKLF7-Reverse	CTGTCGCAGTGGTTACACT
iDraxin-Forward	CAGGGAGGTTTAGGACAAACAG
iDraxin-Reverse	TGTAGGAGCTGAGGGAAAGAAG
PCR	
Cre-Forward	CATATTGGCAGAACGAAAACGC
Cre-Reverse	CCTGTTTCACTATCCAGGTTACGG
KLF7-Forward	ATCAACAGTACAAGCTGAAGGGC
KLF7-Reverse	AACAATGCAAAAGAGGAGATGCC
RT-qPCR	
qGapdh-Forward	CATCACTGCCACCCAGAAGACTG
qGapdh-Reverse	ATGCCAGTGAGCTTCCCGTTCAG
qKLF7-Forward	GTCCATCGCTGTCAGTTTAA
qKLF7-Reverse	TTTCCTGTAGTGCCTTGTGA
qRac3-Forward	ACTCAGCCAACGTGATGGTGGA
qRac3-Reverse	CGGACATTCTCAAAGGAGGCTG
qCdc42ep2-Forward	ATCTCCTTCCAGGAACAGCGGT
qCdc42ep2-Reverse	GAGATGGCGTTTTTGAGCAGAGG
qCdc42-Forward	CAGCGATGAGAAAGATGCCAGAG
qCdc42-Reverse	TCCGACGAACTTTCCAGAGTGG
qDraxin-Forward	GTGGCAGAGAACACAAGAGACG
qDraxin-Reverse	GGTCTTCAGAGGGTTCCACCTT
qEomes-Foward	CCACTGGATGAGGCAGGAGATT
qEomes-Reverse	GTCCTCTGTCACTTCCACGATG
qCwc22-Forward	CCAGAGACAACCCACGCAATAC
qCwc22-Reverse	TGGTTTCTGGGCTACGATGACC
qHapln4-Forward	CGCCAAAATGCCATCCTGTTCG
qHapln4-Reverse	CATAGTGGCACCGTCCTTGATC
qTrim36-Forward	CTATGCGTTCCGAGTGAGAGCT
qTrim36--Reverse	CAGCAAGAGGTGCTCAGTGTTG
qCoch-Forward	GTGTATGCGTCAGTGTCCAGCA
qCoch-Reverse	CGTTGGCATCTACCGAGGAGTA
qScn9a-Forward	GGTCATGGTGATTGGGAACCTTG
qScn9a-Reverse	TCTGGAGGTTGTTTGCGTCGGT
qSyt12-Forward	GACGAGGATGAGCGGAATGTGA
qSyt12-Reverse	GGTAACTGAGGGACAGCAGGAT
qS100a10-Forward	GACAAAGGAGGACCTGAGAGTG
qS100a10-Reverse	CTCTGGAAGCCCACTTTGCCAT

qExtl1-Forward	CAATGCCACCTTCTGCCTCATC
qExtl1-Reverse	ATGATGGCTGCCTTGGTCCAGT
Luciferase reporter assay plasmids	
Luc-Draxin-Forward	TGGCCGGTACCTGAGCTCGCTAGCCTCGAGCACATACAA TAGAGGACTTCCGGATCTTCA
Luc-Draxin-Reverse	GCTTGGCCGCCGAGGCCAGATCTTGATATCGAGCGAGGG GCGGGGACGGCGCGCAGGGAG
Luc-Rac3-Forward	TGGCCGGTACCTGAGCTCGCTAGCCTCGAGTTTCTCTATC GATAGGTACCGCTGAGCCGCATGAAGGCTG
Luc-Rac3-Reverse	GCTTGGCCGCCGAGGCCAGATCTTGATATCCTTAGATCG CAGATCTCGAGGGGCGCGGGGCCGGGCGGCGGCG
**Electroporation plasmids**	
pCAGGS-Draxin-IRES-GFP-Forward	TTTTGGCAAAGAATTCGGCGCGCCATCGATATGGCAGGGTG CCCCGTCCTCAGGGTCCCCCCG CTC GAG
pCAGGS-Draxin-IRES-GFP-Reverse	CGACTGCAGAACTAGAAGCTTGAGCTCGAGCTAGATGTTGA TGAAAGATCCCTGGTCCCC

Development • Supplementary information
